# Research on Time Series-Based Pipeline Ground Penetrating Radar Calibration Angle Prediction Algorithm

**DOI:** 10.3390/s24020379

**Published:** 2024-01-08

**Authors:** Maoxuan Xu, Feng Yang, Yuanjin Fang, Fanruo Li, Rui Yan

**Affiliations:** 1 School of Mechanical Electronic and Information Engineering, China University of Mining and Technology (Beijing), Beijing 100083, China; 2 Beijing Drainage Group Co., Ltd., Beijing 100044, China

**Keywords:** underground space security, pipeline penetrating radar robot, deflection angle prediction, intelligent deflection correction, long short-term memory neural networks

## Abstract

The pipeline ground-penetrating radar stands as an indispensable detection device for ensuring underground space security. A wheeled pipeline robot is deployed to traverse the interior of urban underground drainage pipelines along their central axis. It is subject to influences such as resistance, speed, and human factors, leading to deviations in its posture. A guiding wheel is employed to rectify its roll angle and ensure the precise spatial positioning of defects both inside and outside the pipeline, as detected by the radar antenna. By analyzing its deflection factors and correction trajectories, the intelligent correction control of the pipeline ground-penetrating radar falls into the realm of nonlinear multi-constraint optimization. Consequently, a time-series-based correction angle prediction algorithm is proposed. The application of the long short-term memory (LSTM) deep learning model facilitates the prediction of correction angles and torque for the guiding wheel. This study compares the performance of LSTM with an autoregressive integrated moving average model under identical dataset conditions. The subsequent findings reveal a reduction of 4.11° and 8.25 N·m in mean absolute error, and a decrease of 10.66% and 7.27% in mean squared error for the predicted correction angles and torques, respectively. These outcomes are achieved utilizing the three-channel drainage pipeline ground-penetrating radar device with top antenna operating at 1.2 GHz and left/right antennas at 750 MHz. The LSTM prediction model intelligently corrects its posture. Experimental results demonstrate an average correction speed of 5 s and an average angular error of ±1°. It is verified that the model can correct its attitude in real-time with small errors, thereby enhancing the accuracy of ground-penetrating radar antennas in locating pipeline defects.

## 1. Introduction

Extensive subterranean drainage systems intersect the urban expanse. In the event of a rupture or leakage within a subterranean drainage pipeline, the ground will give way, resulting in the accumulation of sediment within the pipeline and impeding the flow of urban drainage, thereby gravely compromising the safety of urban operations. Within municipal subterranean pipelines, the drainage pipes are customarily positioned at the base of the substructure. The presence of damaged and leaky drainage pipes contributes to the erosion of neighboring soil, leading to the formation of voids surrounding the pipes, and ultimately instigating the collapse of urban thoroughfares. Thus, the assessment of drainage pipe quality and the identification of voids in their vicinity are of utmost importance in the prevention of urban road collapse.

**Figure 1:**
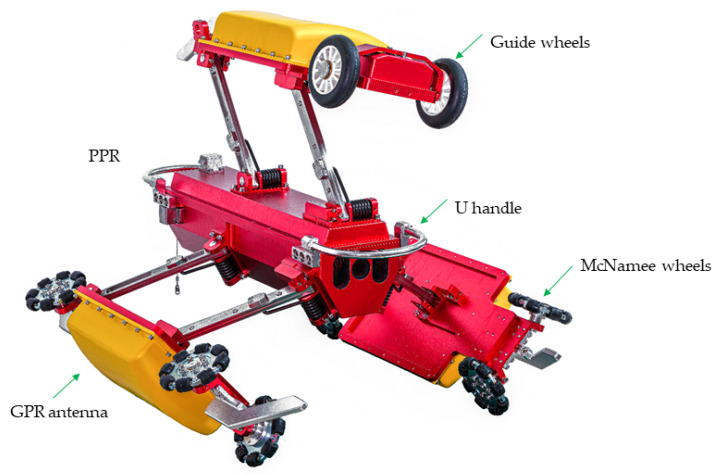
Three-channel drainage pipeline ground penetrating radar device.

The pipeline ground-penetrating radar (PPR) detection device is capable of effectively detecting cavities in the vicinity of the pipeline. This capability represents one of the fundamental technologies employed in urban underground space security systems [1]. The research object of this study is the three-channel drainage pipeline radar detection device [2,3], which has been developed by the China University of Mining and Technology (Beijing) and is depicted in Figure 1. This device consists of a video system and a radar antenna system that are used to determine the corrosion depth of the pipe top and detect voids outside the pipe. Notably, the three channels of the device are positioned at an angle of 120°, enabling simultaneous detection of the pipeline interior, pipe body, and the external environment of the pipeline.

The pipeline GPR is pulled by an electric cable reel at a constant speed to travel inside the drainage pipe. The deviation correction device is located in front of the radar antenna on the top of the PPR. The deviation correction device includes a guide wheel and a servo motor. When the attitude of the PPR deflects, the MCU controls the servo motor to drive the guide wheel to rotate and dynamically correct the attitude angle of the PPR.

The detection of pipeline diseases in engineering employs a traction support wheel for the movement of the PPR, ensuring accurate positioning along the target axis. However, during the actual detection process, the PPR is inevitably influenced by factors such as weight, slope changes, and travel speed, resulting in automatic rotation [4,5,6]. This leads to roll angle deviation, necessitating the correction of the radar antenna angle to accurately locate anomalies in spatial position.

In recent years, scholars and experts have conducted research on PPR attitude control and trajectory planning for correction. For instance, Ren T. et al. compared and analyzed the characteristics of different screw drive in-pipe robots, focusing on mechanism design, driving principles, and motion and mechanical behaviors [7]. The trajectory correction algorithm of an intelligent tunneling robot system is based on fuzzy proportional-integral-derivative (PID), effectively eliminating lateral errors of the road header for trajectory correction [8]. Yeh T. et al. designed four control systems to regulate screw speed, lifting torque, steering angle, and overrun angle, enhancing the performance of the robot’s helical motion in pipes with varying inclinations [9]. However, these methods face challenges in practical applications, including instability, low reproducibility, and the need for extensive training data sets.

The integration of classical control and advancements in deep-learning-based methods has led to the development of new research techniques [10,11,12,13]. Patel R. et al. effectively combined an adaptive sliding mode controller (ASMC) with recurrent neural networks like LSTM and gated recurrent units (GRU) to develop robust adaptive motion control techniques [14]. Chen K. et al. proposed a composite control model that combines PID control closed-loop with an LSTM maneuverability model open-loop, improving the dynamic obstacle avoidance ability of robots [15]. Inoue M. et al. proposed a robot path planning method that combines rapidly exploring random tree (RRT) and LSTM networks, leveraging their high learning and generalization capabilities [16]. Steuernagel L. et al. suggested the use of an encoder–decoder sequence-to-sequence neural network for complex motions where Kalman predictors cannot account for future robot actions, resulting in better motion planning and decision-making [17].

The existing support wheel pipeline inspection equipment is pulled along the pipeline via an electric cable reel, and the attitude angle adjustment of the PPR is achieved by adjusting the angle of the guide wheel. However, this operation accuracy is greatly dependent on the operator, leading to a range of issues such as lengthy correction times and over-correction. Consequently, it becomes challenging to ensure the detection and positioning accuracy of the radar antenna. Hence, it is imperative to explore automatic correction control methods for the PPR.

Section 2 describes the rotation characteristics exhibited by the PPR that have been developed internally. Section 3 offers a demonstration of how the corrective trajectory is generated. Section 4 presents the prediction of the corrective angle, which is based on the LSTM technique. Section 5 showcases the experimental results obtained from the self-developed pipeline radar robot. Lastly, Section 6 draws conclusions for the paper.

## 2. PPR Rotation Characteristics

The PPR is influenced by various factors while moving inside a pipeline, leading to a gradual change in its initial motion attitude. The three sets of walking mechanisms of the PPR are designed spatially at equal intervals of 120°. To ensure the accuracy of the GPR antennae in locating the defects inside the pipeline, it is necessary to keep the cross-roll angle of the PPR stable. And, it should be corrected when the roll angle changes.

For the PPR to travel in a predetermined attitude, it travels along the central axis without deflection. The spin deflection during its travel must be corrected. The method is to apply an attitude correction moment. The vector of moments pointing in the positive direction of the PPR is specified to be positive. A positive correction moment amplifies the PPR’s roll angle.

### 2.1. Attitude Angle of the PPR

**Figure 2:**
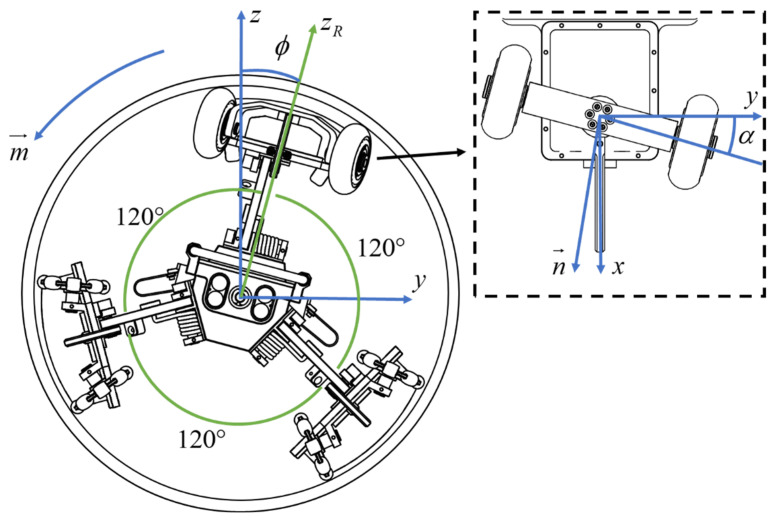
Definition of attitude angle and deflection stress of PPR front view.

The definition of the PPR’s attitude angle and the decomposition of attitude deflection force are illustrated in Figure 2. This figure shows the projection of the PPR’s travel direction in the drainage pipe, with the view angle directly aligned with the radar antenna. The attitude angle (roll angle) ϕ, is defined as the angle between the X–Z plane of the pipeline coordinates and the follower wheel. Counterclockwise rotation is considered positive, while clockwise rotation is considered negative.

In Figure 2, the xyz axes constitute a pipeline coordinate system, where the *x*-axis aligns with the pipeline axis, and the *z*-axis opposes the direction of gravitational force. The $z_{R}$ axis corresponds to the direction of the top radar antenna on the PPR. $\vec{m}$ represents the correction direction in the yz plane, the $\vec{n}$ signifies the forward direction of the PPR. α denotes the angle between the guiding wheel and the pipeline axis, and this angle is utilized to generate the attitude correction angles and torques for the PPR, facilitating its realignment. In this context, the PPR adjusts its attitude correction angles and torques to maintain alignment with the desired direction.

Applying appropriate attitude correction torques to the PPR facilitates the effective adjustment of the radar antenna’s detection angle to meet specific operational requirements. By modulating the magnitude and direction of the attitude correction torque, aligning it with the error in the attitude angle α is achieved.

**Figure 3:**
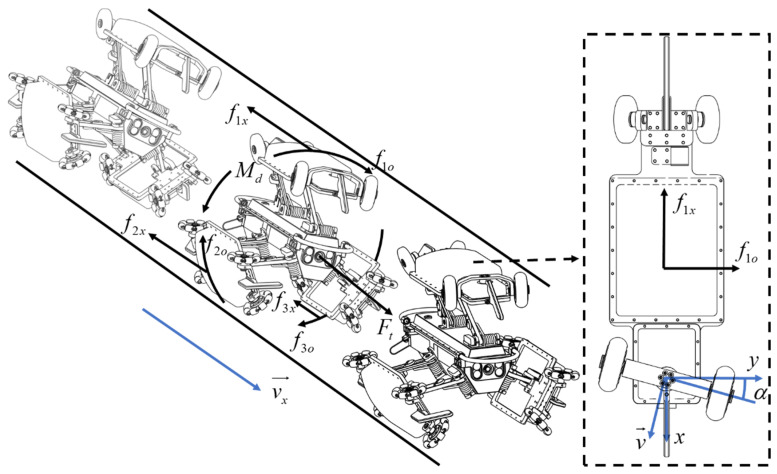
Schematic diagram of guide wheel deflection correction of PPR.

The process of attitude correction for the guiding wheels of the PPR is depicted in Figure 3. In the diagram, the details of the correction mechanism are in the dashed arrow and square. The *v* represents the speed of the PPR along the pipeline axis. Under the influence of the attitude correction torque $M_{d}$, tangential reactive force $f_{io}\left(i=1,2,3\right)$ arises between the support wheels of the PPR and the pipeline wall. The traction force experienced by the PPR is denoted as $F_{t}$, and $f_{ix}\left(i=1,2,3\right)$ represents the frictional force acting along the pipeline axis. This calibration process involves motion velocity, attitude correction torque, and interactions with the pipeline wall to achieve precise control of the PPR.

If the support wheels of the PPR are rigid, three potential scenarios may arise:

1. When the lateral reaction force $f_{io}\left(i=1,2,3\right)$ does not exceed the lateral attachment limit between the support wheel and the inner wall of the pipe.
  (1) $$\sum_{i=1}^{3}f_{io}R=M_{d}<\sum_{i=1}^{3}N_{i}f_{ic}R$$
  where $N_{i}$ is the closure force of the support wheel *i*, $f_{ic}$ is the lateral attachment coefficient between the support wheel and the pipe wall, R is the radius of the pipe. There is no lateral sliding between the support wheel and the pipe wall, the support wheel still travels in its flat direction, and the attitude correction torque $M_{d}$ is suppressed for the correction of the attitude angle.
2. When the lateral reaction force $f_{io}\left(i=1,2,3\right)$ reaches the lateral attachment limit between the support wheel and the inner wall of the pipe.
  (2) $$\sum_{i=1}^{3}f_{io}R=M_{d}=\sum_{i=1}^{3}N_{i}f_{ic}R$$
  Then lateral sliding occurs between the support wheel and the pipe wall; the sliding velocity is $v_{x}$, the support wheel then travels in the direction of the synthetic velocity, the attitude correction torque $M_{d}$ takes effect on the correction of the attitude angle, and after Δt moments, the PPR attitude angle changes to:
  (3) Δϕ=vcΔtR
3. When the attitude correction torque continues to increase by $M_{d}>\sum_{i=1}^{3}N_{i}f_{ic}R$, the PPR attitude angle correction equation is
  (4) $$J_{x}\ddot{\phi }=M_{d}-\sum_{i=1}^{3}N_{i}f_{ic}R$$
  where $J_{x}$ is the rotational inertia of the PPR around its central axis. At this time, the PPR changes its attitude angle from the initial position with angular acceleration.

Since the support wheel of the PPR cannot achieve complete rigidity, when the support wheel is elastic in the lateral direction, even if the lateral reaction force $f_{io}$ does not reach the limit of lateral attachment between the support wheel and the inner wall of the pipeline, the direction of travel of the support wheel will deviate from the pipeline axis. The angle of deviation β is related to the closure force $N_{i}$ on the support wheel and the attitude correction torque $M_{d}$. In the case of a certain closure force $N_{i}$, when the angle of *β* is small, the relationship between $M_{d}$ and β is linear as follows:

(5) $$M_{d}=k\beta$$

where k is the lateral stiffness of the support wheel in N·m/rad. The larger the lateral stiffness, the smaller the lateral elasticity of the support wheel, and the smaller *β* is. Therefore, under the action of attitude correction torque $M_{d}$, the attitude angle formed due to the lateral elasticity of the support wheel is corrected as:

(6) $$\{\begin{array}{l} \Delta \phi =\frac{S_{a}}{R}\tan\beta =\frac{S_{a}}{R}\tan\frac{M_{d}}{k}\\ S_{a}=v_{x}\Delta t \end{array}$$

where $S_{a}$ is the distance moved by the PPR along the pipeline axis; $v_{x}$ is the speed.

In summary, the PPR is analysed by the guiding wheel to generate the attitude correction angle and torque to form a certain angle *α* between it and the pipeline axis, as shown in Figure 3. When the PPR is in the pipeline with a pipeline radius of *R*, moving along the pipeline axis, with a speed *v*, the guiding wheel makes a mixed motion of sliding and rolling. At this time, the friction force along the lateral component perpendicular to the pipeline axis will generate the attitude correction torque, the expression of which is:

(7) $$M_{d}=0.5k_{1}vR\sin2\alpha$$

where $k_{1}$ is the correction torque coefficient [18], and $\alpha =45^{\circ }$ produces the maximum attitude correction torque. By adjusting the size of the angle, the size and direction of the attitude correction torque can be adjusted to correct the PPR’s roll angle.

### 2.2. PPR Motion Resistance

During the movement of the PPR within drainage pipes, the antenna experiences pressure against the pipeline wall due to the action of torsion spring elasticity. If the guiding wheel is subjected to pressure against the pipeline wall, frictional forces will arise between the guiding wheel and the pipeline wall. If the direction of the frictional force forms an angle with the pipeline axis, it generates the necessary corrective torque for attitude correction. The greater this frictional force, the larger the corrective torque. Due to the influence of gravity, pressure between the left and right sections of the radar and the pipeline wall generates frictional forces that impede the torque required for attitude deviation. This frictional force directly hinders the correction of the PPR. An increase in frictional resistance leads to a reduction in the ability to correct at the same deviation angle, and vice versa. These frictional forces are influenced by pressure. Therefore, the pressure between the radar and the pipeline wall plays a crucial role in the correction process. Pressure provides essential parameters for calculating the deviation trajectory.

**Figure 4:**
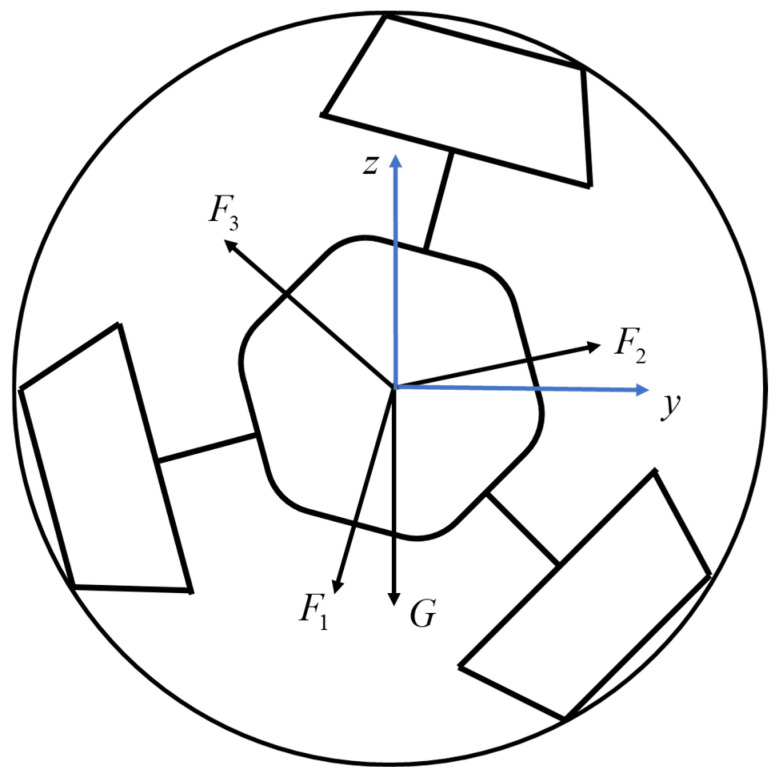
Simplified force diagram of PPR.

The force diagram, as shown in Figure 4, simplifies the PPR in its movement within the pipeline.

PPR experiences gravitational force G; the three arc-shaped radar antennas receive from the pipeline wall support forces $F_{1},F_{2},F_{3}$; the deviation angle of the PPR is ϕ. The equilibrium equation in the yz-plane of the pipeline can be expressed as follows:

(8) $$-F_{1}\cos\phi +F_{2}\sin(30^{\circ }-\phi )+F_{3}\sin(30^{\circ }+\phi )-G=0\;-F_{1}\sin\phi +F_{2}\cos(30^{\circ }-\phi )-F_{3}\cos(30^{\circ }-\phi )=0$$

**Figure 5:**
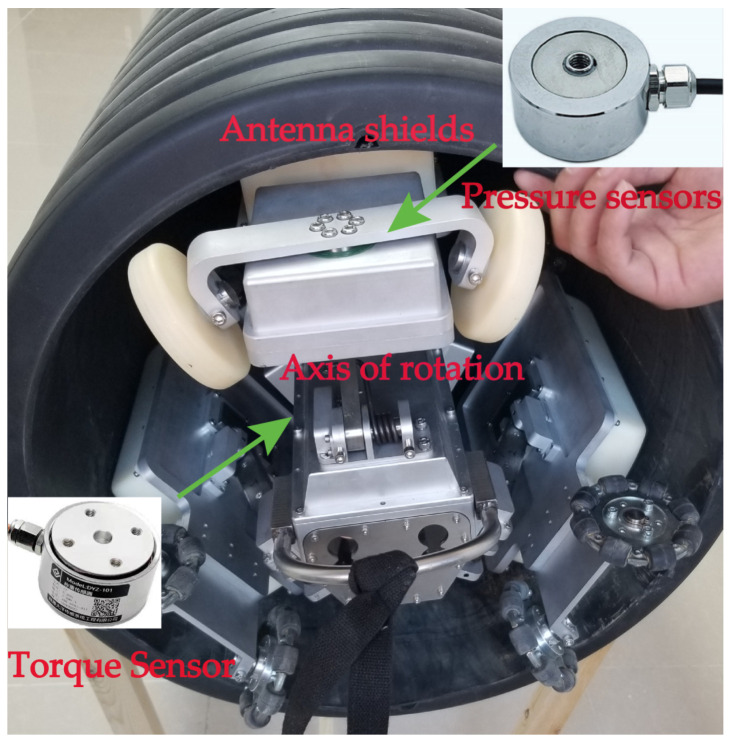
Pressure sensor test.

The PPR motion resistance is mainly through the presetting of pressure sensors on the key components to collect their force changes during the detection process, as shown in Figure 5.

The test chooses to collect the vertical pressure on the cover and the torque of the rotating shaft and pre-set the pressure sensor in the cavity of the cover, respectively. The acceleration value is measured according to the built-in gyroscope. The relationship between pressure and friction can be expressed as:

(9) $$\begin{array}{cc} \mu_{i}F_{i}=f_{i}=\sqrt{f_{ix}^{2}+f_{io}^{2}} & \left(i=1,2,3\right) \end{array}$$

where $f_{i}$ is the frictional force experienced by the radar antenna; $\mu_{i}$ is the friction coefficient between the radar and the pipeline wall. While accurately estimating the friction coefficient may be challenging, the motion resistance of the PPR is closely linked to the pressure exerted by the pipeline wall on the three radar antennas. Therefore, considering pressure as an input parameter for optimizing the deviation trajectory is reasonable.

## 3. Calculation and Optimization of Corrective Trajectories

### 3.1. Calculation of Corrective Trajectories

Based on the above deflection factors of PPR inside the pipeline, the Euler–Lagrange method is used to derive the dynamic equations, and the PPR deflection correction torque can be expressed as:

(10) $$\tau =M\left(q\right)\ddot{q}+H\left(q,\dot{q}\right)+G(q)+\tau_{f}$$

where q, $\dot{q}$ and $\ddot{q}$ are the deflection angle vector, velocity vector, and acceleration vector. M(q) is the inertia matrix of the PPR, $H\left(q,\dot{q}\right)$ is the centrifugal and Coriolis force matrix, G(q) is the heavy torsion matrix, $\tau_{f}$ is the friction vector.

(11) $$\tau_{r}=\Phi \left(q,\dot{q},\ddot{q}\right)\theta +\tau_{f}$$

where $\Phi \left(q,\dot{q},\ddot{q}\right)$ is the observation matrix, which is related only to the deflection angle, velocity, acceleration, and junction structure of the PPR, and θ is the vector of basic inertial parameters, including the mass, center of gravity, and moment of inertia, $\tau_{f}$ given by the calculation in the previous section.

Concerning the accuracy of the PPR’s dynamic model and correction angle prediction, it can be assessed in terms of the root mean square error (RMSE) of the PPR’s predicted angles and torques concerning the actual measured angles and torques. The smaller the RMSE, the better the dynamic model and predicted angles.

### 3.2. Corrective Trajectory Optimisation

The first five terms of the Fourier series are used as the corrective trajectory for the identification and study of the kinetic parameters of the PPR. This periodic function facilitates multiple repetitions of experiments and the transmission of information such as angle, velocity, and torque.

(12) $$q\left(t\right)=q_{0}+\sum_{k}^{5}\left(a_{k}sin\left(k\omega_{f}t\right)+b_{k}cos\left(k\omega_{f}t\right)\right)\;$$

where q(t) is the trajectory function with respect to time *t*, $\omega_{f}$ is the fundamental frequency, $q_{0}$ is the offset constant, $a_{k}$, $b_{k}$, $q_{0}$ and $\omega_{f}$ are the trajectory parameters.

The objective function W is defined by the condition number of the observation matrix:

(13) J=Cond(W)

where W is the ratio of the singular value extremes of the observation matrix Cond(W).

The precision of the rectified trajectory is linked to the noise reduction capability of the observation matrix, and the goal function impacts the sensitivity of the discriminated parameters to the measurement error, which subsequently influences the accuracy of the resultant parameters. The smaller the goal function, the greater the deviation correction effect. Simultaneously, the rectifying trajectory of PPR must also comply with the constraints of the maximum deflection angle, the velocity, and acceleration of the initiation and termination phases. Consequently, the rectifying trajectory can be optimized as follows:

(14) $$\{\begin{array}{c} \min(J)\\ q_{min}\leq \left|q\left(t\right)\right|\leq q_{max}\\ {\dot{q}}_{min}\leq \left|\dot{q}\left(t\right)\right|\leq {\dot{q}}_{max}\\ {\ddot{q}}_{min}\leq \left|\dot{q}\left(t\right)\right|\leq {\ddot{q}}_{max}\\ q(t_{0})=q\left(t_{M}\right)=0\\ \dot{q}(t_{0})=\dot{q}\left(t_{M}\right)=0\\ \ddot{q}(t_{0})=\ddot{q}(t_{M})=0 \end{array}$$

where $q_{min}$, $q_{max}$, ${\dot{q}}_{min}$, ${\dot{q}}_{max}$, ${\ddot{q}}_{min}$, ${\ddot{q}}_{max}$ are boundary values for angle, velocity and acceleration. $t_{0}$, $t_{M}$ are the starting and ending moments.

**Figure 6:**
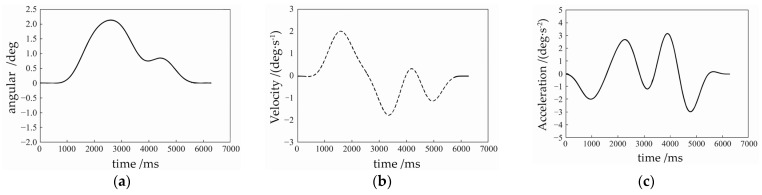
Optimized PPR’s deflection trajectories: (**a**) Angle correction trajectory of PPR; (**b**) Velocity correction trajectory of PPR; (**c**) Acceleration correction trajectory of PPR.

The optimisation of the corrective trajectory is similar to a nonlinear multi-constraint optimisation problem, which can be optimised by objective optimisation algorithms such as Non-dominated Sorting Genetic Algorithms (NSGA) [19] and the optimised PPR angular corrective trajectory is shown in Figure 6a, and the velocity corrective trajectory is shown in Figure 6b, and the acceleration corrective trajectory is shown in Figure 6c. The trajectory is gentle, and the starting and stopping points are all back to the middle, which is not beyond the limitation range, but the optimisation calculation needs to be carried out at each moment, which does not have the memory of past optimisation experiences, and thus the computational resources consumed are larger, and this corrective trajectory is used in the subsequent predictions of the corrective angle and experimental research.

In the actual detection process, the use of NSGA algorithm is not very effective in completing the PPR attitude correction. However, it can be applied to obtain the value of the roll angle under different correction angles.

## 4. Corrective Angle Predictions Based on LSTM Neural Network

The optimisation algorithm in the previous section, due to the need for a large number of iterative calculations at each moment in the process of travelling, is unable to meet the requirements of accuracy and speed simultaneously. In the actual pipeline deskewing process, it needs to be dragged forward at a slower speed due to the poor real-time performance, and the computational accuracy is not sufficiently high, the efficiency is low, and sometimes even non-convergence occurs. However, due to the large amount of data collected in the actual test, different trends of the cross-roll angle can be obtained for different deflection correction angles under different parameter environments. In the following, an LSTM neural network model will be used to train the existing actual data in order to simplify the process of calculating with a large number of iterations at every time, and to substantially improve the deflection correction speed and accuracy, and to predict the deflection of the pipeline.

For the characteristics of PPR deflection in drainage pipes, a total of 8000 sample sets are constructed based on different roll angles, slope changes, torque, and travelling speeds, as shown in Table 1.

### 4.1. LSTM Neural Networks

For serialized data, Recurrent neural network (RNN) processing method can be used. LSTM network is based on this, for the joint action of historical and new information, and targeted improvement. LSTM can thus learn the features of time series better and avoid the problem of gradient vanishing [20,21]. Multiple structural units are connected to form the unit structure of LSTM, which extends with time and depth, and can improve the problem of insufficient memory and the slow computation of objective optimization algorithms such as NSGA.

**Figure 7:**
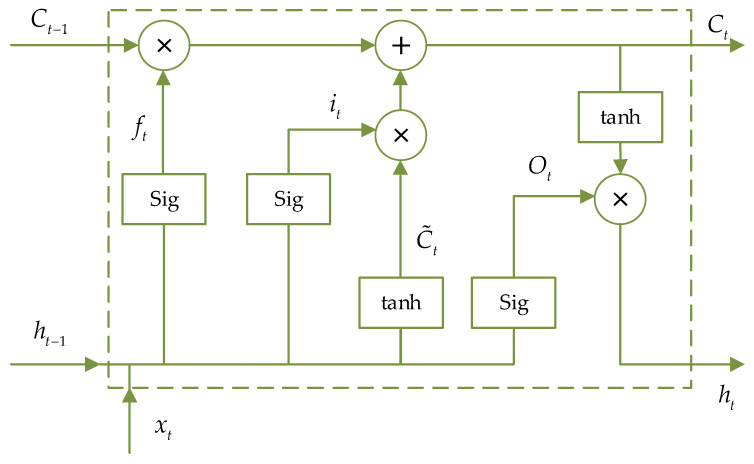
Long short-term memory cell of hidden layer.

The motion process of the PPR in the pipeline, and the dynamics data during its motion, is a typical continuous time series. The time-varying factors, such as friction, cause the attitude change of the PPR, which fits well with the characteristics of LSTM. In this study, LSTM cells with cell states and gate structure are used to improve the prediction effect:

(15) $$\left\{ \begin{array}{c} i_{t}=Sig\left(W_{i}\cdot \left[h_{t-1},x_{t}\right]+b_{i}\right)\\ o_{t}=Sig\left(W_{o}\left[h_{t-1},x_{t}\right]+b_{o}\right)\\ {\tilde{C}}_{t}=\tanh\left(W_{C}\cdot \left[h_{t-1},x_{t}\right]+b_{C}\right)\\ C_{t}=f_{t}*C_{t-1}+i_{t}*{\tilde{C}}_{t}\\ f_{t}=Sig\left(W_{f}\cdot \left[h_{t-1},x_{t}\right]+b_{f}\right)\\ h_{t}=o_{t}*\tanh\left(C_{t}\right) \end{array}\right.$$

where $x_{t}$ is the input vector at the current time *t*. $h_{t-1}$ is the output of the preceding LSTM network at the current time. $W_{i}$, $W_{o}$, $W_{C}$, $W_{f}$ are the weight matrices of the input gate, output gate, cell unit and forgetting gate of the previous LSTM network at the current time *t*. $b_{i}$, $b_{o}$, $b_{C}$ and $b_{f}$ are the corresponding offsets. *C* and *h* are the output vector of the cell and the implied state, respectively. i and $\tilde{C}$ are the intermediate variables of cell. o denotes intermediate variables of the output gate. The cell structure of the hidden layer of the LSTM neural network is shown in Figure 7.

The training method for LSTM networks is backpropagation through time (BPTT), which is similar to the BP algorithm and consists of steps such as forward computation, backward computation, calculating the gradient and updating the parameters. There are many types of gradient-based optimization algorithms, the Adaptive moment estimation (Adam) algorithm [22], which is used to optimize the LSTM network training method, can compute the adaptive learning rate for different parameters and occupies less storage resources. Compared to other stochastic optimization methods, the Adam algorithm performs better in most practical applications [19].

### 4.2. LSTM Corrective Angle Prediction Process

The composition of the dataset for the LSTM network is centered around the deflection angle. This dataset encompasses a total of 8000 datasets. The six distinct categories within this dataset include mileage, slope, deflection angle, velocity, acceleration, and deflection torque of the PPR in the drain. The dataset is constructed using input data collected from sensors carried by the PPR in the drain, as well as the outcomes of the deflection correction within the dataset. Of the entire dataset, 80% is allocated as the training set for deflection correction, while the remaining 20% is designated as the test set data.

**Figure 8:**
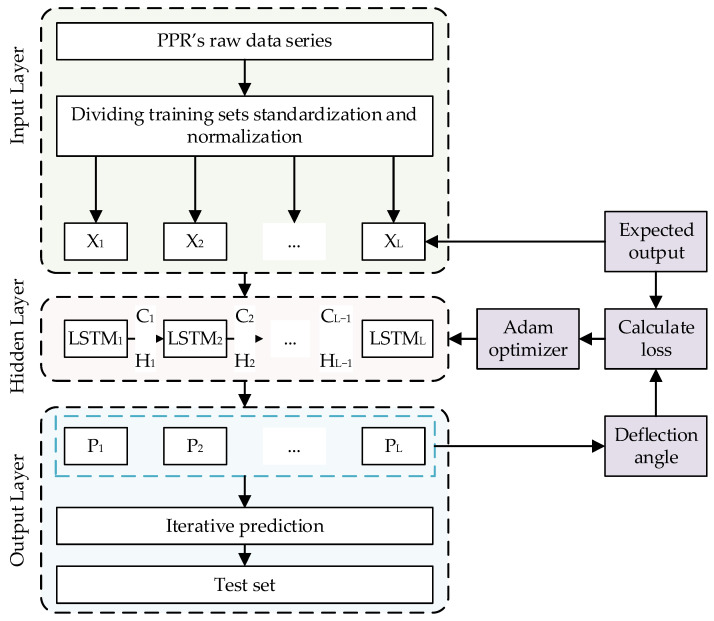
Flowchart of PPR deviation angle prediction based on LSTM network.

The LSTM-network-based PPR deviation angle prediction process is shown in Figure 8. It including: the LSTM network, the dataset generation and the network training method. The input and output layers of the LSTM network are fully connected networks and the hidden layer consists of LSTM units. The X is the distribution data, C is the cell output and H is the cell hidden state, the main steps are as follows:

1. Torque and corrective angle prediction using the original data being dynamically modelled;
2. Normalization of the training set and test set raw data, including predicted torque, actual torque and actual position;
3. Training set into the fully connected input layer;
4. Iterative computation in the hidden layer;
5. Predicted torque and corrective angle of the LSTM are given through the output layer;
6. Calculate the loss, the RMS error of the predicted torque and kink angle with respect to the actual measured torque and kink angle;
7. Perform network parameter optimization through Adam optimizer;
8. Weight update is performed and applied;
9. The test set calculates the compensated predicted torque and deflection correction angle through the network and performs the prediction effect evaluation.

Based on the trial calculations and the actual situation, the parameters of the pre-trained model network structure for PPR corrective angle prediction are obtained, as shown in Table 2. The input vectors are the calculated torque, the actual torque, the actual speed and the actual angle at each moment of history. The output vectors are the predicted torque and corrective angle at the next moment, and the number of nodes of the LSTM network is selected according to the feature dimensions, and the loss is the root-mean-square error of the predicted angle and torque at the current moment relative to the predicted angle and torque at the next moment.

## 5. Correction Angle Experiment

### 5.1. Composition of PPR Deviation Correction Mechanism

**Figure 9:**
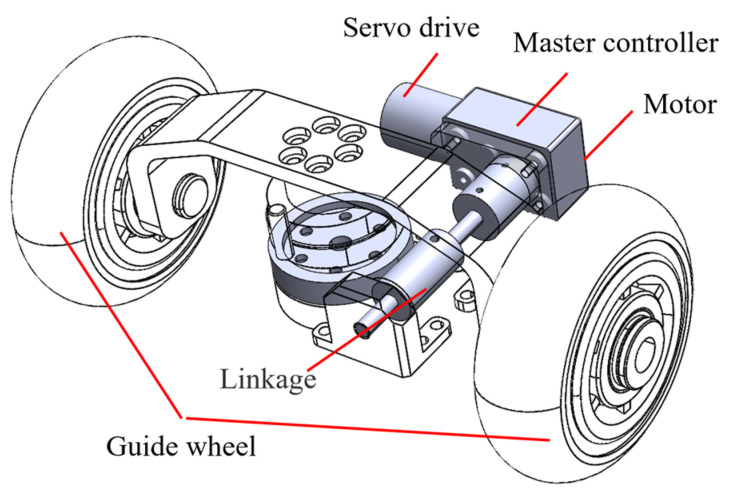
Correction mechanism of PPR.

The three-channel PPR guiding mechanism in the drainage pipe mainly consists of the main controller, motor driver, motor, guide wheel and drive shaft, as shown in Figure 9. The main controller is an ARMv7-based MCU, and the servo driver is a dual H-bridge FOC controller that supports EtherCAT high-speed and low-latency communication.

The servo drive is set to cyclic synchronised position mode. The servo drive and the main controller communicated via EtherCAT, with the controller sending serial points of the excitation trajectory in the graph to the drive, using a fixed frequency of 100 Hz to ensure continuous and smooth motion. While the three-channel PPR in the drainage pipe is in motion, the main controller also communicates with the drive to collect data such as angle, speed and torque.

**Figure 10:**
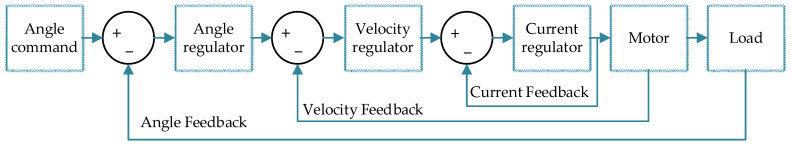
Overall control scheme of motor.

The utilization of a triple-loop structure control algorithm, which is frequently employed for servo motors, is implemented due to the absence of influence on the intrinsic characteristics of the PPR. These three loops consist of a current loop, a velocity loop, and an angle loop, progressing from the innermost layer to the outermost layer. These aforementioned control loops can be categorized as PID controllers, which utilize feedback signals. The holistic motor control design is visually represented in Figure 10.

### 5.2. PPR Correction Experiment

**Figure 11:**
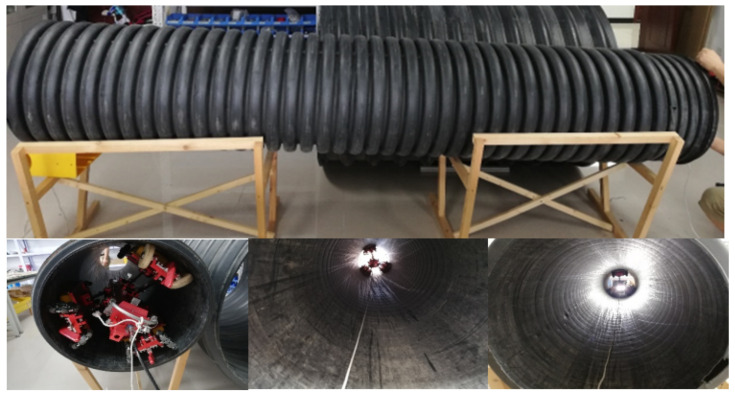
Sample diagram of PPR deviation correction experiment.

Through conducting 20 iterations of PPR correction experiments on a series of pipelines, a total of 8000 raw data points were acquired, each with a time interval of 1 s. Among these data points, 80% were allocated to serve as correction training sets, while the remaining 20% were designated as test set data. The training set consists of the optimized correction trajectory, while the test set encompasses the correction trajectory that has been subjected to additional constraints. A visual representation of the experimental site can be observed in Figure 11.

The corrective trajectories and experimental data are used as training data for the LSTM network by predicting the angle and torque at the current moment from the velocity and acceleration at the previous moment. The root-mean-square error of the theoretical computed angle and torque and the actual angle and torque are used as the loss function.

$$Loss=\sqrt{\frac{1}{N}\sum_{i=1}^{N}{\left({\hat{y}}_{i}-y_{i}\right)}^{2}}$$

where ${\hat{y}}_{i}$ is the anticipated value of the correction angle as predicted by the model, $y_{i}$ is the measured value of the correction angle in the experiment.

**Figure 12:**
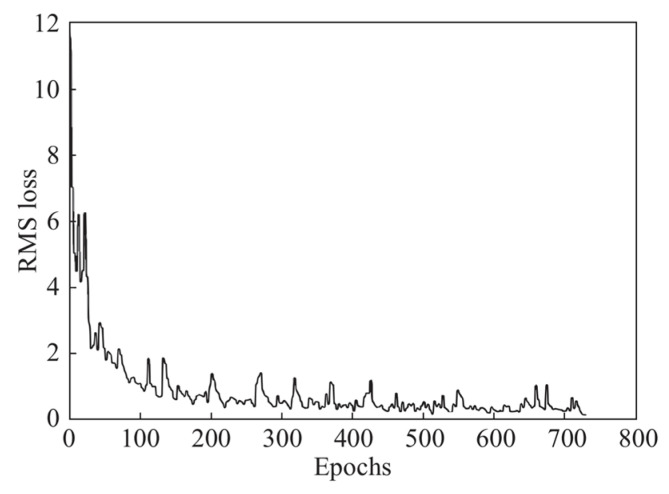
Epochs vs. RMS loss.

As the number of iterations of the LSTM network increases, the loss gradually decreases, and when the iteration reaches about 800 times, the decreasing speed and fluctuation of the loss tends to level off, and the training is terminated at this time. The relationship between the number of iterations and the loss is shown in Figure 12.

**Figure 13:**
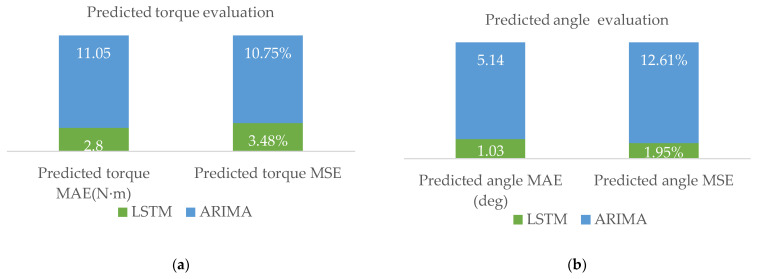
Comparison of evaluation indicators: (**a**) Predicted angle MAE and MSE; (**b**) Predicted torque MAE and MSE.

A comparison of the prediction results of the angle and torque by LSTM and the traditional differential Autoregressive Integrated Moving Average (ARIMA) model in the same data set is shown in Figure 13, and the two error functions are used to calculate the Mean Absolute Error (MAE) and Mean Square Error (MSE) between the measured and predicted values to evaluate the performance indexes of the intelligent deviation correction prediction model. These expressions are as follows:

(16) $$MAE=\frac{1}{N}\sum_{i=1}^{N}\left|{\hat{y}}_{i}-y_{i}\right|$$

(17) $$MSE=\frac{1}{N}\sum_{i=1}^{N}{\left({\hat{y}}_{i}-y_{i}\right)}^{2}$$

where ${\hat{y}}_{i}$ is the normalized predicted value, $y_{i}$ is the normalized measured value, N is the number of samples in the training set.

**Figure 14:**
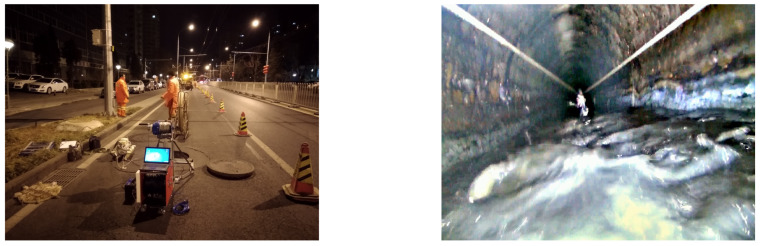
The PPR in drainage pipe intelligent deflection correction experiment.

The trained neural network model is arranged in the MCU firmware of the PPR and is used for real time attitude correction in the drainage pipe. The PPR is pulled by the motor of the wheel at a constant speed as it travels in-pipe. The concrete pipe diameter is 600 mm, length is 4.5 m, water content of 1/3, and wall containing silt. The experimental environment is shown in Figure 14. The mean values of 10 sets of experimental data are shown in Table 3.

The experimental findings indicate that under identical dataset conditions compared to ARIMA, the LSTM demonstrates a reduction in MAE for predicting yaw angles and torque by 4.11° and 8.25 N·m, respectively. The MSE also shows a decrease of 10.66% and 7.27%. When contrasted with ARIMA and conventional PID control methods, the average correction time is 5 s with an average angular error within ±1°. The experimental results validate its correction capability within these error margins. The LSTM predictive model enhances the real-time responsiveness and accuracy of PPR’s attitude correction.

## 6. Conclusions

The self-developed PPR proposed in this study demonstrates a structure that is lightweight and possesses a minimal force of inertia. However, the trajectory of the robot is disrupted by various factors, including motion resistance, travelling speed, slope change, and human factors, which result in deviations from the target axis during traversal. In order to tackle the problem of low accuracy and significant uncertainty associated with the traditional manual angle correction manipulation, this study introduces a correction angle compensation method based on LSTM network. This method effectively reduces the error between the predicted and actual angles.

The experimental results reveal that the proposed approach, in comparison to the ARIMA model, achieves a higher level of accuracy, as indicated by the lower MAE and MSE values. Therefore, the corrective angle compensation technique based on the LSTM network is proven to be both feasible and effective, serving as a solid foundation for future research in dynamic control. Nevertheless, there are a few technical challenges that persist during the actual implementation, such as the optimization of the network structure of the prediction model, which necessitate further investigation in subsequent studies.

## Figures and Tables

**Table 1:** Sample set of PPR’s deflection angles.

Parameters	Training Set	Test Set	Sum
Mileage (cm)	4000	1000	5000
Slope (deg)	320	80	400
Angle (deg)	1600	400	2000
Acceleration (m/s^2^)	80	20	100
Torque (N·m)	350	50	400
Velocity (m/s)	80	20	100

**Table 2:** Parameters of pre-training model for correction angle prediction of PPR.

Parameters	Value
LSTM cell layer	3
Time step	1
Batch size	16
Input size	2
Output size	2
Learning rate	0.05

**Table 3:** Comparison between LSTM, ARIMA and PID methods for experimental data.

Parameters	Average Corrective Time (s)	Average Angular Error (deg)
LSTM	5.05	±1.42
ARIMA	7.12	±3.06
PID	9.53	±6.14

## Data Availability

Data are contained within the article.
